# A Simplified Protocol for Tracheostomy Decannulation in Patients Weaned off Prolonged Mechanical Ventilation

**DOI:** 10.1055/s-0043-1776720

**Published:** 2024-02-05

**Authors:** K. Devaraja, C. S. Majitha, Kailesh Pujary, Dipak Ranjan Nayak, Shwethapriya Rao

**Affiliations:** 1 Department of Otorhinolaryngology and Head and Neck Surgery, Kasturba Medical College, Manipal Academy of Higher Education, Manipal, Karnataka, India; 2 Department of Critical Care Medicine, Kasturba Medical College, Manipal Academy of Higher Education, Manipal, Karnataka, India

**Keywords:** tracheostomy, mechanical ventilation, ventilator weaning, laryngoscope, swallowing, airway extubation

## Abstract

**Introduction** The criteria for the removal of the tracheostomy tube (decannulation) vary from center to center. Some perform an endoscopic evaluation under anesthesia or computed tomography, which adds to the cost and discomfort. We use a simple two-part protocol to determine the eligibility and carry out the decannulation: part I consists of airway and swallowing assessment through an office-based flexible laryngotracheoscopy, and part II involves a tracheostomy capping trial.

**Objective** The primary objective was to determine the safety and efficacy of the simplified decannulation protocol followed at our center among the patients who were weaned off the mechanical ventilator and exhibited good swallowing function clinically.

**Methods** Of the patients considered for decannulation between November 1st, 2018, and October 31st, 2020, those who had undergone tracheostomy for prolonged mechanical ventilation were included. The efficacy to predict successful decannulation was calculated by the decannulation rate among patients who had been deemed eligible for decannulation in part I of the protocol, and the safety profile was defined by the protocol's ability to correctly predict the chances of risk-free decannulation among those submitted to part II of the protocol.

**Results** Among the 48 patients included (mean age: 46.5 years; male-to-female ratio: 3:1), the efficacy of our protocol in predicting the successful decannulation was of 87.5%, and it was was safe or reliable in 95.45%. Also, in our cohort, the decannulation success and the duration of tracheotomy dependence were significantly affected by the neurological status of the patients.

**Conclusion** The decannulation protocol consisting of office-based flexible laryngotracheoscopy and capping trial of the tracheostomy tube can safely and effectively aid the decannulation process.

## Introduction

Tracheostomy is the creation of an alternate airway in the form of a tracheocutaneous fistula by bypassing the upper airway. It is performed to provide an alternate pathway for breathing in patients with upper airway obstruction or to facilitate prolonged mechanical ventilation in patients who are neurologically unstable or present with respiratory insufficiency. However, most of the tracheostomies performed for the aforementioned indications are temporary. Once the inciting factor is tackled, the tracheostomy tube is removed to restore the normal physiological breathing through the larynx, pharynx and nose. This process of removal of the tracheostomy tube is known as decannulation. Although the procedure itself may sound simple, the implications of improperly-performed and abrupt decannulation can be catastrophic at times. 1 2

To be eligible for decannulation, a patient with a tracheostomy should have a reasonable neurological status, with a good swallowing function, an adequate airway, and a satisfactory pulmonary function. 3 4 5 6 To elaborate, as for the neurological status, the patient should be conscious and alert, and have a good cough reflex to clear the secretions from the lower airway. They should be able to demonstrate well-coordinated swallowing without any apparent aspiration. Regarding the airway, there should not be any significant narrowing in the upper airway that could offer resistance to nasal breathing. Lastly, the patient's pulmonary function should be satisfactory, with adequate pulmonary reserve and enabling good oxygen exchange. These parameters must be assessed in any tracheostomized patient before they are considered for the decannulation process. In addition to the aforementioned major criteria, the underlying indication for the tracheostomy must also have been resolved, and it is preferable to have all procedures requiring general anesthesia in this regard prior to the decannulation. 7

Despite the consensus on the operational principles of any decannulation protocol, the actual investigative method used to assess the necessary parameters before decannulation varies significantly between centers. 8 9 10 For instance, while several medical centers ask for a computed tomography scan of the neck and thorax to assess the adequacy of the airway before decannulation, a few others prefer direct endoscopy of the airway under general anesthesia to decide on the eligibility for decannulation. 11 12 Similar differences among the centers can also be observed with regards to their methods to assess swallowing function prior to decannulation. 13 14 15 16 While these methods may seem reliable, their cost-effectiveness remains questionable, and the added risk of radiation exposure or general anesthesia keeps them from being included in the routine clinical practice. At our center, we follow a simple and clinically-feasible protocol for decannulation that is devoid of any such major investigations. In the present study, we have evaluated the safety and efficacy of this simplified decannulation protocol in a peculiar cohort of tracheostomized patients.

## Materials and Methods

### Study Design and Setting

The primary objective of the present retrospective study was to determine the safety and efficacy of a simplified decannulation protocol followed at our center for the of temporary tracheostomy among the patients with non-obstructive indications. A secondary analysis was performed to identify if any factors, such as age, gender, duration of tracheostomy dependence, and indication for tracheostomy could have any bearing on the decannulation outcomes.

### Study Subjects

All patients considered for decannulation by the Department of Otorhinolaryngology between November 1st, 2018, and October 31st, 2020 were evaluated to be included in this study. The primary selection criterion was elective surgical tracheostomy performed to facilitate prolonged mechanical intubation (and weaning off ventilatory support). In other words, the patients who had undergone tracheostomy for upper airway obstruction, secondary to a neoplasm, deep neck space infection, or trauma to the neck, were not included in the present study. In addition, patients younger than 15 years of age and those decannulated within a week of undergoing tracheostomy were also excluded.

### Decannulation Protocol

The algorithm of our decannulation protocol contains two parts. In part I, which involves an assessment of the airway and swallowing, an office-based transnasal flexible laryngoscopy is used to evaluate the lumen of the upper airway, vocal cord mobility, and aspiration-free swallowing. For the swallowing assessment in particular, we perform a trial of oral feeds with colored liquids or semi-solids and observe for any aspiration by flexible laryngoscopy. For the patients who demonstrate aspiration during this evaluation, the decannulation protocol shall be deferred until a few sessions of swallowing therapy and clearance on a formal fiberoptic endoscopic evaluation of swallowing (FEES) on a later date. The factors deemed favorable in flexible laryngoscopy to proceed with decannulation include aspiration-free swallowing, mobility of at least one vocal cord, and absence of any gross narrowing of the lumen, as assessed subjectively by at least one of the eight consultant otorhinolaryngologists of the department. In patients who exhibit favorable parameters in the upper airway, consequently, a flexible tracheoscopy is performed through the tracheostoma (after the removal of the tube) to assess the status of the trachea, both below (antegrade – until the carina) and above (retrograde – until the subglottis) the stoma. In many of our patients, a plain radiograph of the neck is also used at this point to objectively assess the adequacy of the airway, particularly if there are any elements of doubt in the endoscopic transnasal or transstomal assessment. If the airway is deemed compromised (other than the minimal subglottic edema), either by subjective assessment on laryngoscopy or objective assessment on radiograph, that patient no longer proceeds with the decannulation protocol, and is subjected to further management of the airway narrowing. At our center, to reduce the interobserver variability, we use a standardized assessment protocol, covering the aforementioned parameters and respective adequacy criteria for each parameter.

**Fig. 1:**
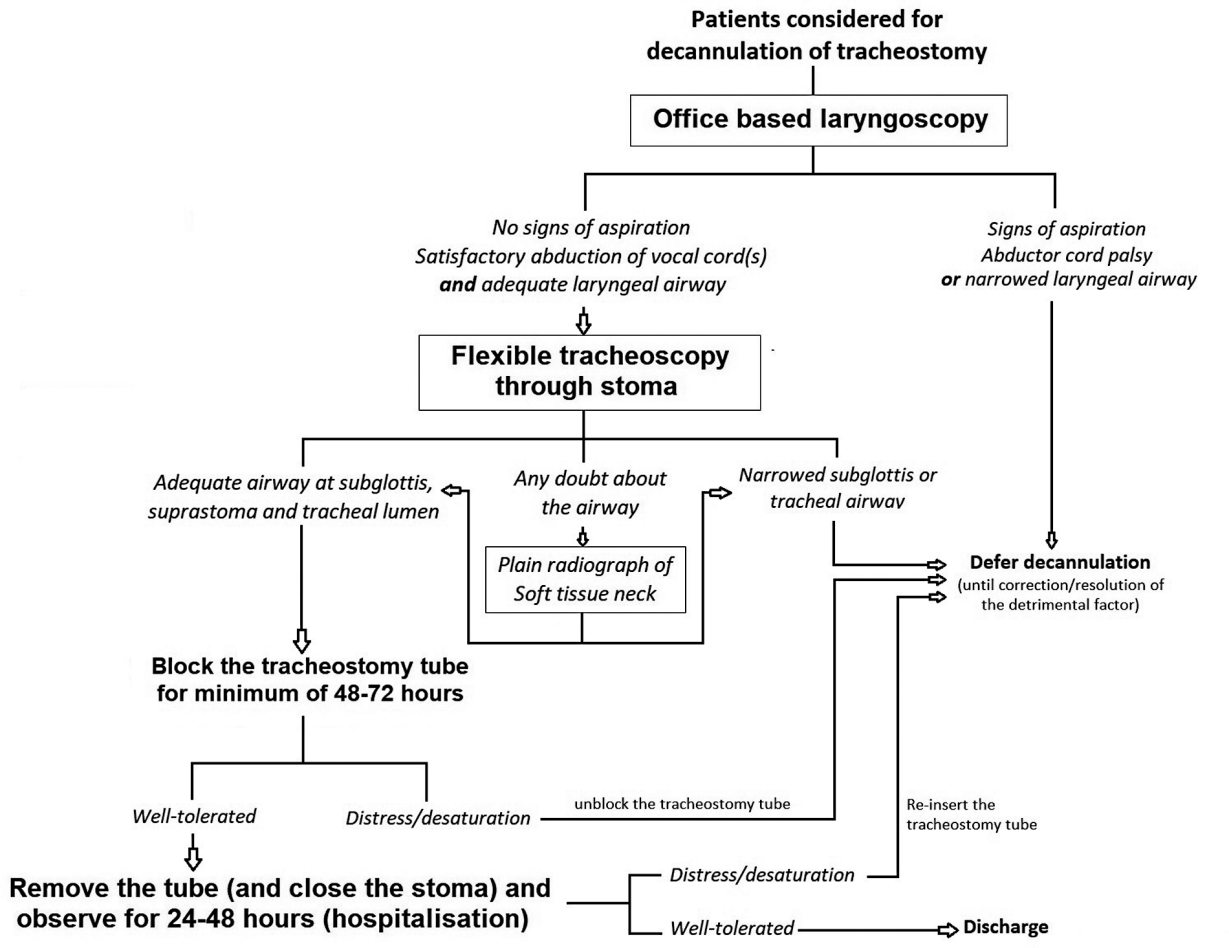
Algorithm depicting simplified protocol used at our center for tracheostomy decannulation.

In patients who fulfill these criteria, a trial of corking the tracheostomy tube is performed as part II of the protocol: the tracheostomy tube is blocked either after downsizing or on a fenestrated tracheostomy tube (cuff deflated) and monitored for any breathing difficulty or signs of aspiration. Once the patient can tolerate the corked tracheostomy tube for a minimum of 48 to 72 hours, without any respiratory distress or swallowing issues, the tube is removed, and a small dressing is applied over the tracheocutaneous fistula, enabling it to close on its own in a few days. During this step, called strapping, some of the warning signs to look for are cough or breathing difficulty while swallowing, tachycardia, desaturation, and noisy breathing. At our center, this step of strapping is performed as an in-patient procedure, with continuous monitoring of the breathing pattern, pulse rate, and blood oxygen saturation by oximetry for at least 24 hours. The schematic algorithm of this simplified decannulation protocol is depicted in Fig. 1 .

### Data Collection

The clinical records of the eligible patients were retrieved from the hospital medical records, and the necessary details were collected and tabulated on a spreadsheet. The parameters recorded in each case consisted of demographics, in the form of age and gender, associated comorbidities, indication for tracheostomy, duration of the tracheostomy dependence, evaluation prior to decannulation, and outcome of the decannulation. Subsequently, the patients were divided according to their outcomes into one group that only underwent part I and another group that underwent both parts of the protocol.

### Outcome Analysis

The success rate of the decannulation among the patients who had been deemed eligible to undergo part I of the protocol was taken as the efficacy of our protocol to predict the decannulation success. The safety profile of the protocol was defined as its ability to correctly predict the chances of risk-free decannulation without the need for revision tracheostomy among the patients who tolerated the corking trial. In other words, for the final analysis, overall efficacy = C/A and safety profile or reliability = C/B, in which A is the total number of patients deemed fit for decannulation after part I (assessment), and B is the total number of patients who tolerated corking and were deemed fit for decannulation after part II (corking trial), and C is the total number of patients who were decannulated successfully without any distress or revision tracheostomy within a month of the decannulation.

For the secondary objective, the clinicopathological factors were analyzed by univariate and multivariate analyses to assess if any of those variables played a significant role in predicting successful decannulation. All the statistical analyses were performed using the IBM SPSS Statistics for Windows (IBM Corp., Armonk, NY, United States) software, version 20.0.

## Results

### Patient Characteristics

A total of 81 tracheostomy patients underwent a decannulation attempt during the study period, 48 of whom fulfilled the eligibility criteria and were included for further analysis. Among the excluded patients were one child, seven cases of upper aerodigestive tract malignancy, five cases of deep neck space abscess, eight cases of bilateral abductor cord palsy, three posttracheoplasty cases, six patients of trauma to the neck, and three due to incomplete medical records. The mean age of the included patients was of 46.5 (range: 15 to 78) years, and the male-to-female ratio was of 3:1. Of all the patients who had undergone tracheostomy to facilitate prolonged mechanical ventilation and weaning off the ventilator support, 22 had an underlying neurological illness as the indication for tracheostomy. The various neurological conditions among these cases were cerebrovascular accident ( *n* = 7), traumatic brain injury ( *n* = 6), Guillain-Barré syndrome ( *n* = 3), and miscellaneous ( *n* = 6). The 26 remaining patients were on prolonged mechanical ventilation for non-neurological conditions, such as respiratory insufficiency due to organophosphorus poisoning ( *n* = 14), severe pneumonia ( *n* = 5), acute respiratory distress syndrome due to various causes ( *n* = 4), sepsis secondary to acute necrotizing pancreatitis ( *n* = 1), acute renal failure ( *n* = 1), and scrub typhus ( *n* = 1). The clinical characteristics of the included patients have been summarized in Table 1 .

**Table 1:** Clinical characteristics of our study cohort

Patient ID	Age (in years)	Sex	Indication for tracheostomy	Duration of tracheostomy (in weeks)	Results of decannulation
SDP-1	41	Female	Cerebrovascular accident	11	Failed corking
SDP-2	60	Male	Aspiration pneumonia	8	Successful
SDP-3	49	Male	Acute respiratory distress syndrome	2	Successful
SDP-4	46	Male	Organophosphorus poisoning	3	Recannulated
SDP-5	64	Male	Organophosphorus poisoning	4	Successful
SDP-6	74	Male	Cerebrovascular accident	19	Successful
SDP-7	72	Male	Pulmonary nocardiosis and pneumonia	3	Successful
SDP-8	47	Male	Organophosphorus poisoning	3	Successful
SDP-9	55	Female	Cerebrovascular accident	9	Successful
SDP-10	15	Male	Traumatic brain injury	8	Successful
SDP-11	68	Male	Traumatic brain injury	8	Failed corking
SDP-12	51	Male	Acute respiratory distress syndrome	11	Successful
SDP-13	61	Male	Vascular dementia	12	Successful
SDP-14	54	Male	Acute respiratory distress syndrome	7	Successful
SDP-15	50	Male	Organophosphorus poisoning	3	Successful
SDP-16	26	Female	Wernicke encephalopathy and axonal neuropathy	9	Failed corking
SDP-17	63	Male	Traumatic brain injury	5	Successful
SDP-18	33	Male	Organophosphorus poisoning	3	Successful
SDP-19	53	Female	Ventilator-associated pneumonia	5	Successful
SDP-20	71	Male	Organophosphorus poisoning	5	Successful
SDP-21	25	Male	Quadriparesis	19	Successful
SDP-22	62	Male	Tetanus	11	Successful
SDP-23	38	Male	Organophosphorus poisoning	2	Successful
SDP-24	25	Male	Organophosphorus poisoning	4	Successful
SDP-25	23	Male	Traumatic brain injury	9	Recannulated
SDP-26	22	Male	Traumatic brain injury	24	Successful
SDP-27	54	Male	Cerebrovascular accident	8	Failed corking
SDP-28	71	Male	Acute inflammatory demyelinating polyneuropathy	53	Successful
SDP-29	38	Female	Acute kidney Injury	8	Successful
SDP-30	30	Male	Organophosphorus poisoning	4	Successful
SDP-31	32	Male	Guillain-Barré syndrome	26	Successful
SDP-32	78	Male	Cerebrovascular accident	15	Successful
SDP-33	60	Female	Acute necrotizing pancreatitis	6	Successful
SDP-34	26	Male	Organophosphorus poisoning	17	Successful
SDP-35	22	Female	Cerebrovascular accident	4	Successful
SDP-36	47	Female	Acute respiratory distress syndrome	7	Successful
SDP-37	22	Male	Organophosphorus poisoning	3	Successful
SDP-38	49	Female	Scrub typhus	3	Successful
SDP-39	61	Female	Bulbar palsy	26	Successful
SDP-40	54	Male	Cerebrovascular accident	13	Successful
SDP-41	51	Female	Organophosphorus poisoning	4	Successful
SDP-42	62	Male	Guillain-Barré syndrome	3	Successful
SDP-43	20	Male	Traumatic brain injury	7	Successful
SDP-44	73	Male	Global dysfunction with respiratory muscle weakness	32	Successful
SDP-45	52	Male	Organophosphorus poisoning	15	Successful
SDP-46	44	Male	Acute pneumonia	2	Successful
SDP-47	24	Female	Organophosphorus poisoning	5	Successful
SDP-48	16	Male	Guillain-Barré syndrome	10	Successful

NOTE: SDP = Simplified decannulation protocol.

### Primary Outcome

All of the included patients had been deemed eligible for decannulation after part I of the protocol, and they were subjected to part II (A = 48). However, four of these patients did not tolerate the process of corking despite being declared as fit for decannulation (B = 44). Of the remaining 44 who had been decannulated after favorable response in both parts I and II, two patients experienced distress after discharge and had to undergo revision tracheostomy within two weeks of the decannulation process (C = 42). Statistically, the protocol was safe or reliable in 95.45% of the decannulated patients, and the overall efficacy in predicting successful decannulation was of 87.5%.

### Secondary Outcomes

As for the predictive factors for successful decannulation, as shown in Table 2 , in both univariate and multivariate analyses, the outcome was affected by the underlying indication for tracheostomy. The patients with non-neurological illnesses demonstrated a statistically higher probability of being decannulated than those with a neurological comorbidity. The average duration of tracheostomy dependence was of ten weeks. Post hoc, when the duration of tracheostomy dependence was analyzed separately as per the underlying indication, the mean duration was significantly higher ( *p* = 0.005 by the Student *t* -test) for the group with neurological illnesses (14 weeks) as compared to those with other conditions (6.5 weeks).

**Table 2:** Factors predicting the success of decannulation in the study cohort

Factors	Variables	Univariate	Multivariate***
Age	Continuous variable	0.99 *	0.83
Gender	Male versus Female	0.43 **	0.59
Duration of tracheostomy	Continuous variable	0.05 *	0.22
Indication for prolonged ventilation	Neurological versus non-neurological	0.005 **	0.002

Notes: *Student *t* -test. **Chi-squared test. ***Multinomial logistic regression.

## Discussion

In 2012, a group of multidisciplinary experts convened by the American Academy of Otolaryngology - Head and Neck Surgery (AAO-HNS) published a clinical consensus guideline on tracheostomy care, including prerequisites and steps for decannulation. 7 According to this consensus guideline, to be eligible, a patient should have adequate consciousness, reasonable laryngopharyngeal function (with at least one mobile vocal cord), and a good cough reflex to facilitate aspiration-free swallowing and to protect the lower airway. In addition, the fiber-optic laryngoscopy should confirm the adequate airway until the immediate subglottis, and, before the removal of the tracheostomy tube, the patient should tolerate capping without any distress or discomfort. The protocol herein described is also based on the AAO-HNS guideline. However, to improve its safety profile, as a routine practice before the decannulation, we additionally assess the airway above and below the tracheostoma through flexible tracheoscopy. Flexible tracheoscopy helps identify luminal lesions of the upper trachea, which can go undetected by laryngoscopy and could preclude the process of decannulation. 17 18 19 As per a report, 19 in up to 20% of the patients who tolerated tracheostomy capping, flexible tracheoscopy enabled appropriate management of luminal lesions and avoided the risk of decannulation failure and revision tracheostomy. Moreover, performing the retrograde and antegrade tracheoscopy after removing the tube could also aid in unmasking the underlying tracheomalacia, which can be observed in many patients with long-standing tracheostomy dependence. 1

The assessment of the airway by flexible tracheoscopy is dependent on the observer. 19 In the present study, although eight consultant otolaryngologists were involved in the airway assessment, an evaluation of the interobserver variability was not possible due to the retrospective nature of the study. However, we believe that the use of a standardized assessment (with fixed parameters and a predefined adequacy criteria for each parameter) in the present study has ensured a good interobserver agreement. Moreover, to avoid undervaluation of the airway by flexible tracheoscopy, whenever required, an additional plain radiograph of the neck was performed at our center to objectively obtain the necessary information about the airway adequacy. The plain radiograph could also be useful to assess the airway before decannulation in uncooperative adults and children. Compared with cross-sectional imaging, such as computed tomography, or the direct assessment of the airway under general anesthesia, which entail the unnecessary exposure of supposedly vulnerable patients to either radiation or general anesthesia, a combination of flexible tracheoscopy and plain radiograph of the neck can be a simpler and clinically-reliable alternative to assess the airway that may have a wider clinical usefulness. Similarly, though there are numerous complex algorithms to assess swallowing function prior to decannulation, the overall yield of such procedures may not justify their routine use in the clinical practice, particularly in developing nations. 14 15 As reiterated by the results of the present study, a simple transnasal flexible laryngoscopy seems to be sufficient to confirm an excellent swallowing function, with preserved laryngeal sensation and cough reflex, even in neurologically-ill patients. 6 However, our decannulation protocol is meant to be used only in patients who exhibit good swallowing function clinically (those accepting oral feeds well), and the method used is not meant to rate or assess the severity of aspiration or to provide corresponding prognostication of decannulation in patients with swallowing issues. For the latter, a formal FEES or videofluoroscopic swallow study would be a better modality. As for the cost-effectiveness, although no approach-to-approach analysis could be performed in the present retrospective study, by taking into account the estimated costs of the investigations involved, such as office-based flexible laryngoscopy (cost = x), plain radiograph (cost = 0.5x), FEES (cost = 2x to 3x), computed tomography (cost = 4x) and evaluation under general anesthesia (cost = 15x to 20x), our simplified decannulation protocol seems to reduce the unwarranted financial burden in the majority of the patients, who otherwise are clinically fit to undergo decannulation. The cost involved in the process of strapping, which may vary between the public (cost = 0.5x to 2x) and private centers (cost = 5x), is not essentially an added burden in our protocol, as this step is generally performed as in-patient procedure in most of the protocols used in other centers. 6 7

Capping is another crucial part of our protocol. While many of the previously published reports support capping before the actual decannulation, a few authors have downplayed its effectiveness in their subjects. 3 17 20 Nevertheless, 42 out of 44 patients who had tolerated capping in the present study experienced successful decannulations, without any respiratory distress or need for reinsertion of the tube. The results of the present study reiterate that capping can reasonably predict the chances of successful decannulation and should be performed in all cases prior to strapping. Capping enables the stimulation of nasal breathing and ensures the maintenance of the tracheostomy tract that can be readily put to use in case of respiratory distress. 8 Apart from exercising the patients' ability to overcome the resistance offered by the upper airway to nasal breathing, capping also tests their ability to clear the secretions from the trachea through the upper airway. 8 Inability to tolerate capping (that is, desaturation or respiratory distress) should indicate ineligibility for decannulation and should prompt a further detailed evaluation to identify the cause. 17

The simplified decannulation protocol herein described showed an efficacy of 87.5% and a reliability of 95.45% in successfully decannulating tracheostomized patients with non-obstructive indications. These results are comparable with those the other methods described earlier, but our protocol is likely to be more cost-effective and feasible. 11 12 13 14 15 16 Around 4.5% of our patients had failed decannulation attempts, despite having been deemed fit for decannulation. The rate of failure, defined as the need to reinsert an artificial airway within a short while of being decannulated after successfully clearing a decannulation protocol, in the present study is similar to those reported in the literature. 4 21 Since most of those who fail a decannulation attempt do so in the first 24 to 48 hours of strapping, we insist on undertaking the process of strapping in the hospital and under supervision. 4 21

Our cohort's average period for decannulation was comparable with the literature reports. 18 20 Regarding the predictors of decannulation, neurological disease as an indication for mechanical ventilation/tracheostomy negatively affected the outcome of decannulation in our study. However, the literature is marred with contradicting reports in this regard. Few studies suggest the negative impact of neurological illness on decannulation success, and a few others refute any association between the indication for tracheostomy and the outcome of decannulation. 19 22 A similar dilemma can be witnessed in the literature when it comes to the association of the decannulation outcome with the age or gender of the patient. 22 23 Similarly, tracheostomies placed for periods shorter than ten weeks have also been reported 16 to increase the chances of successful decannulation; however, the duration of the tracheostomy did not affect the decannulation outcomes in the cohort of the present study. These variations in the literature can be attributed to the differences in study methodology and constituent study subjects.

The limitations of the present study include the absence of a comparison group, as we did not use a control group, or compare the effectiveness or usefulness of alternate methods of swallowing or airway assessment, such as FEES, videofluoroscopy, and computed tomography, respectively. Also, the present study demonstrated the safety and effectiveness of this simple decannulation protocol only in patients who presented with a good swallowing function clinically and did not consider those with poor swallowing function, neither those with airway narrowing. Further studies including subjects with mild to moderate swallowing issues or marginal airway narrowing might be able to determine the usefulness of the protocol in those patients. Lastly, although it would have been interesting to have noted the associations between the decannulation results and the type and location of the neurological lesion, the relevant data to do so was not available for every patient, which hindered such an analysis in the present study.

## Conclusion

To conclude, the present study analyses the outcome of a simple decannulation protocol in a retrospective cohort of tracheostomized patients, in whom the tracheostomy was performed to wean off from the mechanical ventilator. The results indicate that, in a carefully-selected group of patients (with no airway narrowing and good swallowing function clinically), a simple decannulation protocol with office-based flexible laryngoscopy and tracheoscopy through the stoma with or without a plain radiograph of the neck can effectively aid the decannulation process.
